# Beta event-related desynchronization as an index of individual differences in processing human facial expression: further investigations of autistic traits in typically developing adults

**DOI:** 10.3389/fnhum.2013.00159

**Published:** 2013-04-25

**Authors:** Nicholas R. Cooper, Andrew Simpson, Amy Till, Kelly Simmons, Ignazio Puzzo

**Affiliations:** ^1^Centre for Brain Science, Department of Psychology, University of EssexColchester, UK; ^2^Centre for Integrative Neuroscience and Neurodynamics, School of Psychology and Clinical Language Sciences, University of ReadingReading, UK

**Keywords:** alpha, beta, mu, EEG, ERD, autism, emotion

## Abstract

The human mirror neuron system (hMNS) has been associated with various forms of social cognition and affective processing including vicarious experience. It has also been proposed that a faulty hMNS may underlie some of the deficits seen in the autism spectrum disorders (ASDs). In the present study we set out to investigate whether emotional facial expressions could modulate a putative EEG index of hMNS activation (mu suppression) and if so, would this differ according to the individual level of autistic traits [high versus low Autism Spectrum Quotient (AQ) score]. Participants were presented with 3 s films of actors opening and closing their hands (classic hMNS mu-suppression protocol) while simultaneously wearing happy, angry, or neutral expressions. Mu-suppression was measured in the alpha and low beta bands. The low AQ group displayed greater low beta event-related desynchronization (ERD) to both angry and neutral expressions. The high AQ group displayed greater low beta ERD to angry than to happy expressions. There was also significantly more low beta ERD to happy faces for the low than for the high AQ group. In conclusion, an interesting interaction between AQ group and emotional expression revealed that hMNS activation can be modulated by emotional facial expressions and that this is differentiated according to individual differences in the level of autistic traits. The EEG index of hMNS activation (mu suppression) seems to be a sensitive measure of the variability in facial processing in typically developing individuals with high and low self-reported traits of autism.

## Introduction

The study presented here was undertaken in order to examine the usefulness of measuring EEG sensorimotor reactivity to examine individual differences in emotional facial processing. For half a century, it has been known that suppression of the dominant resting rhythm in the EEG over sensorimotor areas accompanies not only movement execution but also movement observation (Gastaut, 1952; Gastaut and Bert, 1954). This rhythm, most commonly known as mu (but also referred to as the Rolandic or wicket rhythm) has two contributing bandwidths: an 8–12 Hz component oscillating at alpha frequencies and a 12–20 Hz low beta band component, perhaps reflecting contributions from primary somatosensory cortex and motor cortex, respectively (Hari, 2006; Avanzini et al., 2012). A substantial amount of experimental work has established that movement execution is associated with suppression of the mu oscillatory activity over the sensorimotor cortex: at rest, the mu bandwidths show a synchronized activity, leading to high-amplitude oscillations. This synchronized activity is functionally distinguishable from the dominant occipital alpha activity. When a movement is executed, this synchronized activity is suppressed and this suppression is thought to reflect active processing in sensorimotor areas (Pfurtscheller and Lopes da Silva, 1999). Such suppression is often referred to as desynchronization or event-related desynchronization (ERD), particularly when it is measured in relation to a pre-stimulus baseline (or reference) period (Pfurtscheller and Aranibar, 1977).

Gastaut and colleagues' investigation of mu activity demonstrated that not only did mu desynchronize to movement execution but also to imagining and observing movements (Gastaut, 1952; Gastaut and Bert, 1954). The findings pertaining to movement observation were under-explored for several decades until the discovery of so-called “mirror neurons” in monkeys in the 1990's (Di Pellegrino et al., 1992; Rizzolatti et al., 1996). Research then turned to looking for human analogs of mirror neurons using various neuroimaging and other psychophysiological techniques. Mirror neurons were originally described as cells in monkey area F5 (an analog of the inferior frontal gyrus in humans and also later in parietal lobule) that fire not only when the animal makes a specific movement but also when it observes that movement (Rizzolatti and Craighero, 2004). Work in humans using fMRI (e.g., Iacoboni et al., 1999, 2005; Molnar-Szakacs et al., 2006), transcranial magnetic stimulation (TMS; Fadiga et al., 1995; Enticott et al., 2010; Sartori et al., 2012), depth electrode recording (Mukamel et al., 2010), and EEG/MEG (e.g., Hari et al., 1998; Nishitani and Hari, 2000; Muthukumaraswamy and Johnson, 2004a,b; Kilner et al., 2009) have since shown the existence of a similar observation-execution matching system that has been labeled the human mirror neuron system (hMNS) as this does not necessitate the existence of “mirror neurons” per se in humans, just a functionally similar mechanism. In this context, it is the EEG/MEG research that has drawn on the work of Gastaut and colleagues to explore the links between mu suppression and the hMNS. Not only has mu-suppression been shown to be a useful indicator of action-observation pattern matching (in that suppression accompanies both action-execution and action-observation) but that it also closely matches other measures of the putative hMNS. For instance, mu-suppression to the observation of hand movements has been shown to closely mirror fMRI BOLD activation in areas analogous in humans to mirror neuron areas in primate studies (Perry and Bentin, 2009). In this context, mu-suppression has also been shown to be modulated by the laterality of the presentation stimulus (i.e., it is driven by the side of the screen on which an observed movement occurs), to be consistent with the reactivity of mirror neurons in area F5 in monkeys (Kilner et al., 2009) and to be dynamically modulated similarly in both action observation and action performance (Press et al., 2011). Accordingly, mu-suppression during action observation is interpreted as an index of activity in the hMNS (Pineda, 2005, 2008; Kilner et al., 2009). Indeed, whereas until recently, mu-suppression during action-observation has been thought to result from post-synaptic modulation from mirror neurons in premotor cortex (Rizzolatti and Craighero, 2004; Pineda, 2008), recent evidence of so-called “M1 view” cells in primary motor cortex with mirror neuron-like properties (Dushanova and Donoghue, 2010) suggests that mu-suppression may be a more direct measure of hMNS than was previously believed, as M1 may itself be a part of the hMNS (Press et al., 2011).

The notion of a hMNS has been used as an argument for the biological mechanisms underlying theories of embodied cognition such as simulation theory. Simulation theory posits that we understand the behaviors and emotions of others by activating similar neural processes in ourselves to those at play in the person observed (Gallese and Goldman, 1998; Gallese, 2009). This has been particularly investigated in relation to how we understand the facial expressions of others. Many studies have found fMRI evidence for common neural activation during both the execution and perception of facial expressions, particularly in areas associated with the hMNS (e.g., Carr et al., 2003; Leslie et al., 2004; Hennenlotter et al., 2005; van der Gaag et al., 2007). This has been strengthened by TMS studies showing that performance on a facial emotion processing task correlates with TMS-induced motor evoked potentials (thought to be an index of hMNS activity; Enticott et al., 2008) and that disrupting pre-SMA activity with TMS impairs the recognition of happy faces (Rochas et al., 2012). To date, although it has been known for some time that mu suppression is sensitive to oro-facial movements (Muthukumaraswamy et al., 2004a), little work has been carried out using EEG to gauge mu reactivity to facial emotion processing. However, a handful of studies report findings that suggest that the use of mu suppression may be useful in this context. For instance, Moore et al. (2012) report mu ERD to both happy and disgusted faces, with an earlier response to disgust and a longer, more extensive response to happy faces. Similarly, decreased beta power (akin to increased beta ERD) has been observed to painful stimuli during the observation of emotional compared to neutral expressions (Senkowski et al., 2011). One other study has also reported a difference between beta reactivity over central electrodes (sensorimotor areas) to angry and happy faces; with increased beta power in the angry condition (Guntekin and Basar, 2007). In addition, Pineda and Hecht have shown that mu suppression is positively correlated with a social-perception task (matching facial expressions based on the eye region alone) but not with a social-cognitive task (judging intentions and beliefs of others), suggesting that the hMNS may be involved in the former behavior but not the latter (Pineda and Hecht, 2009).

With regard to action observation, the use of EEG to measure mu suppression has been useful in terms of discovering clinical and individual differences in sensorimotor (and possible hMNS) activation. Clinically both schizophrenia (McCormick et al., 2012) and autism (Oberman et al., 2005; Bernier et al., 2007) have been associated with abnormal mu reactivity, although much debate remains regarding the robustness and interpretation of these results (Raymaekers et al., 2009; Fan et al., 2010; Puzzo et al., 2011). In terms of individual differences, the level of expertise (Behmer and Jantzen, 2011), amount of learning (Marshall et al., 2009), and degree of habituation (e.g., in smokers; Pineda and Oberman, 2006) have been shown to affect mu suppression. Sex differences have also been observed (Cheng et al., 2008; Silas et al., 2010), along with altered mu reactivity according to the degree of empathy (Perry et al., 2010; Woodruff et al., 2011; Cooper et al., 2012) and the level of autistic traits (Puzzo et al., 2010). However, to date, no studies looking at mu reactivity to facial emotion processing have found any individual differences. Of the three studies to look in this area, two did not investigate individual differences (Guntekin and Basar, 2007; Senkowski et al., 2011) and one, investigating the influence of the level of empathic traits, found no differences between those scoring high and low for empathy (Moore et al., 2012). Given the lack of research in this area and the evidence for the usefulness of mu suppression as an index of individual differences in action observation mechanisms, we undertook to explore its application for investigating the neural mechanisms of facial emotion processing. Specifically, we were interested in examining whether emotionally charged facial expressions (positive, negative, and neutral) modulate the sensorimotor reactivity induced by hand movement observation. In addition, given the debate in the autism literature, we were interested in testing whether or not this reactivity would vary according to the level of self-reported autistic traits in typically developing adults. The benefits of using such a population include, the availability of larger numbers of potential participants, the lack of certain possible confounds such as medication and the potential to gain insight into the boundaries of the disorder (Hirsch and Weinberger, 2003). Indeed, in the last decade, autism spectrum disorder (ASD) classifications have changed, so that now, facets of autism are seen as an extreme end of the behavioral traits observed in the normal population (Baron-Cohen et al., 2001; Constantino and Todd, 2003, 2005). Thus, investigating autistic traits in a typically developing population is useful both for the insight it may provide into autism per se and also into how these traits are manifest in the population as a whole.

## Method

### Participants

Initially, 80 participants completed the Autism Spectrum Quotient (AQ) (Baron-Cohen et al., 2001). From this sample, two groups were formed comprising of 10 high scorers (high AQ group; seven female) and 10 low scorers (low AQ group; six females). The high AQ group was comprised of those scoring ≥22 and the low AQ group scoring <11 (Almeida et al., 2010). Thus, the number of participants in the EEG part of the study was 20 (mean age = 25.4 years). The mean AQ score was 23.9 (*SD* = 2.28) for the high group and 7.6 (*SD* = 1.43) for the low group. All participants gave written informed consent and the study was approved by the University of Essex Ethics Committee.

## Materials

The AQ was used to assess the degree to which adults from a normal population have traits typically associated with ASD (Baron-Cohen et al., 2001). The questionnaire comprises of 50 questions, each item in the AQ scores one point if the participant's answer is an autistic-like answer. Participants' scores can range from 0 to 50, with higher scores associated with high traits of autism.

**Figure 1:**
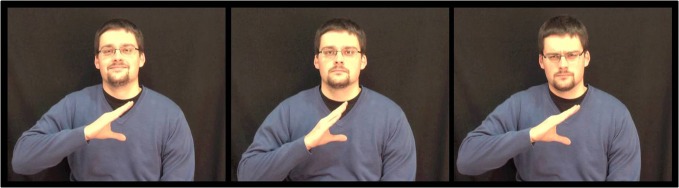
**Stills taken from stimulus video of one actor portraying from left to right: happy, neutral, and angry facial expressions**.

This experiment was part of a larger study looking at social gestures, and for the purposes of this experiment, videos containing actors opening and closing their right hands with three different facial expressions were used (see Figure 1). For each condition (happy, neutral, angry), four actors were filmed (two female) wearing dark clothes against a dark back-drop, and seated in the center of the screen. The actors' hands were held in front of their chests so that both the hand movement and the facial expression were clearly visible. The actors opened and closed their hands at a rate of 1 Hz, holding their fingers and thumbs straight. Thus, in total, there were 12 different video clips that constituted one block. Six blocks were run in total with the presentation of the video clips randomly ordered at the start of each block. Each video lasted 3 s with a 3 s inter-trial interval. Stimuli were presented using Superlab software (Cedrus Corporation, San Pedro, CA) on an Apple PowerMac (2 GHz PowerPC G5; Apple Inc., Cupertino, CA).

### EEG data acquisition

EEG data were recorded with Neuroscan 4.4 acquisition software and SynampsII amplifiers using a 64 channel Quick-Cap arranged according to the international 10–10 system (Compumedics, Melbourne, Australia). Eye movements were recorded using teo facial electrodes—above and below the left eye. Impedances for all electrodes were reduced to below 10 kOhm before the start of each session. All data were continuously sampled at 1000 Hz with a bandpass filter of 0.15–200 Hz and a 50 Hz notch filter. Online EEG data were referenced to a point midway between Cz and CPz, and grounded midway between Fz and FPz.

### EEG data preparation

Following visual inspection of the data, noisy data blocks were rejected. Bad electrodes were excluded on a participant by participant basis (electrode C2 was excluded from one high AQ participant and one low AQ participant; electrode Oz was excluded from three high AQ participants). Ocular artifact rejection was carried out using the Neuroscan Edit transform (derived from Semlitsch et al., 1986) followed by a second, automatic artifact rejection sweep, with exclusion parameters set at ±75 mV. In order to calculate event-related desynchronization/synchronization (ERD/S), the data were epoched from −1500 to 3500 ms around the start of each video clip and the following steps were performed using the event-related band-power transform in Neuroscan Edit 4.4 (Compumedics, Melbourne, Australia): the data underwent complex demodulation and concurrent filtering (zero phase-shift, 24 dB roll-off, envelope computed) into the EEG bandwidths of interest: alpha (8–12 Hz) and low beta (12–20 Hz). It was trimmed (1000 ms from each end, to remove filter warm-up artifacts) and averaged. A reference interval of −500 to 0 ms was used to calculate the percentage change between the active period (500–2500 ms) and it, using the classic method adapted from Pfurtscheller and colleagues (e.g., Pfurtscheller and Aranibar, 1977; Pfurtscheller and Lopes da Silva, 1999): ERD% = (R−A) / R × 100, where R = power in the reference interval and A = power in the active or task phase. Thus, desynchronization and synchronization are expressed as a percentage of activity relative to the reference interval (NB, using this formula ERD produces positive scores and ERS negative). In order to reduce the number of multiple comparisons, the electrodes were collapsed within each hemisphere, resulting in two variables: left central (C5, C3, C1) and right central (C6, C4, C2).

### Design

This experiment was a mixed factor design with two repeated-measures factors: emotional expression (happy, neutral, angry) and hemisphere (left, right) and one between-subjects factor: AQ group (high AQ, low AQ). In order to check that our findings were due to mu activity (i.e., deriving from sensorimotor areas) and not related to occipital alpha we also employed Oz as a control site. For Oz data, there was only one repeated measures factor (emotional expression). The dependent variables for all ANOVAs were the ERD/S values in the alpha and low beta bandwidths. Thus, two mixed measures ANOVAs were carried out for each scalp location (central alpha, central low beta, occipital alpha, and occipital low beta). In order to explore interactions, planned comparisons used one-way ANOVAs to examine between subjects differences and paired students' *t*-tests for repeated measures differences.

## Results

### Central sites (C5, C3, C1, C2, C4, C6)

#### Low beta band

**Figure 2:**
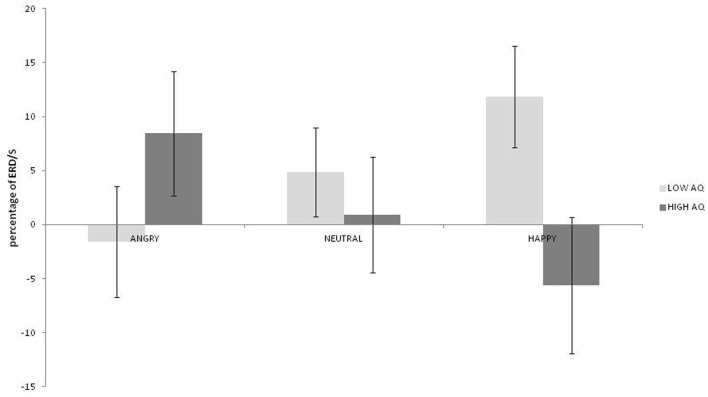
**Low beta ERD percentage-change over central sites for low and high AQ groups during angry, neutral, and happy conditions (positive values indicate ERD, negative scores indicate ERS)**.

No main effects for emotion, hemisphere or group were observed (*ps* > 0.187). A strong interaction was observed between emotion and group [*F*_(2, 36)_ = 9.38; *p* = 0.001; η^2^_*p*_ = 0.343]. As can be seen in Figure 2, this was driven by greater low beta ERD to happy than both angry and neutral expressions in the low AQ group [*t*_(9)_ = 2.867; *p* = 0.019; 95% CI = 2.83 to 24.04 and *t*_(9)_ = 3.327: *p* = 0.009; 95% CI = 2.22 to 11.69, respectively] and by greater low beta ERD to angry than to happy expressions in the high AQ group [*t*_(9)_ = 2.497; *p* = 0.034]. There was also significantly more low beta ERD to happy faces for the low than for the high AQ group [*t*_(18)_ = 2.221; *p* = 0.039; 95% CI = 0.94 to 34.02]. No other two- or three-way interactions were significant (*ps* > 0.154).

#### Alpha band

**Figure 3:**
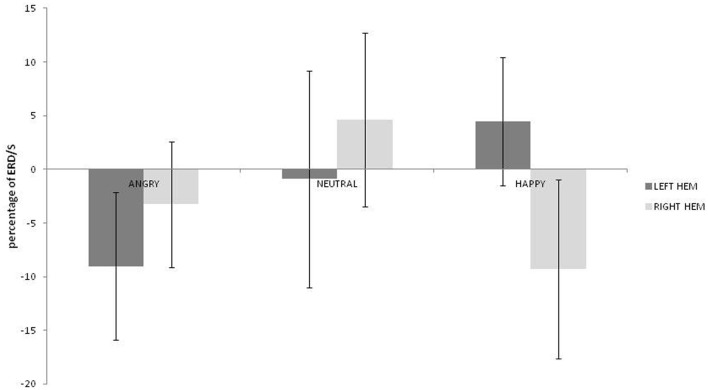
**Alpha ERD percentage-change over central sites for left and right hemispheres during angry, neutral, and happy conditions (positive values indicate ERD, negative scores indicate ERS)**.

No main effects for emotion, hemisphere or group were observed (*ps* > 0.459) but there was a significant interaction between emotion and hemisphere [*F*_(2, 36)_ = 3.492; *p* = 0.041; η^2^_*p*_ = 0.162]. As can be seen in Figure 3, greater alpha ERD was observed for happy than for angry expressions in the left hemisphere [*t*_(19)_ = 2.847; *p* = 0.01; 95% CI = 3.57 to 23.4]. Also, for happy expressions, alpha ERD was greater in the left than in the right hemisphere [*t*_(19)_ = 2.51; *p* = 0.021; 95% CI = 2.28 to 25.26].

### Occipital site (Oz)

Data from three participants (all high AQ group) were omitted due to noise on the Oz electrode. No main effects or interactions were observed in either bandwidth (*ps* > 0.071). This suggests that our findings for the central sites were indeed due to mu activity and not to occipital alpha.

## Discussion

This study sought to examine the usefulness of mu suppression when investigating individual differences in emotional facial processing. Specifically, we investigated whether alpha and low beta ERD over sensorimotor areas would differ according to both the degree of autistic traits of the observer and the facial expression of the observed subject (i.e., the person “doing” the actions). Our main finding was that in the low beta band from central sites (overlying primary motor areas), whereas those scoring high in autistic traits (high AQ group) showed greater low beta ERD to angry compared to happy expressions, those with low AQ scores showed the opposite effect (greater ERD to happy than either angry or neutral expressions). Also, the low AQ group had greater low beta ERD to happy faces than the high AQ group. In the context of action observation, mu suppression is regarded as a reliable index of hMNS activation (Muthukumaraswamy and Johnson, 2004b; Pineda, 2005, 2008; Kilner et al., 2009). In the present study, mu suppression to action observation was modulated by the facial expression of the actor making the hand movement. Consequently, our results suggest that those with higher levels of autistic traits have greater hMNS activation to negative facial expressions (anger) and those with low levels have greater hMNS activation to positive ones (happy). Additionally, when viewing happy expressions, the low AQ group showed greater hMNS activation than the high AQ group. This differentiation according to the level of autistic traits may also help to explain the discrepancy in findings in the previous studies examining mu reactivity in facial processing (Guntekin and Basar, 2007; Moore et al., 2012) as such individual differences were not taken into account in these studies.

It is interesting, and perhaps surprising, that we did not find any differences between AQ groups in the alpha bandwidth or indeed, much in the way of alpha ERD to the stimuli presented, regardless of AQ group. Many previous studies, investigating action observation have shown alpha to be suppressed during the observation of movement (e.g., Muthukumaraswamy and Johnson, 2004a,b; Oberman et al., 2007; Perry and Bentin, 2009) and some have reported differences in this suppression between people with autism and control groups in alpha (Oberman et al., 2005; Bernier et al., 2007). This alpha suppression is typically interpreted in terms of the internal simulation of the movement in the observer. The reason for our lack of findings in this bandwidth is unclear. It is possible that the nature of the stimuli presented may have altered the response (e.g., the relatively small area of the visual scene taken up by the moving hand). Also, with the inclusion of the emotional faces, there is more to take in and potentially more to simulate. It may be that the addition of faces to the stimuli usually presented in such protocols (i.e., moving hands) has a differential modulating effect on the two mu components (alpha and low beta) and that would suggest a different functional role for them both in the simulation process. For instance, it has been suggested that changes in alpha may reflect activation of primary somatosensory cortex, whereas those in beta might indicate motor cortex activity (Hari, 2006; Avanzini et al., 2012) and therefore the results from the current study might reflect relatively greater motor cortex and less somatosensory activation in response to the stimuli. The differential functions of the mu bandwidths in action observation and emotional recognition is an interesting question that merits further investigation.

Returning to our main results in the lower beta band, a superficial interpretation might lead one to expect that those scoring high for autistic traits should be worse at recognizing happy faces (possibly as a result of less emotional resonance with positive emotions). However, a recent meta-analysis of emotional facial processing in autism suggests that while there may be a difficulty in recognizing emotions in autism, recognition of happiness is only marginally impaired (Uljarevic and Hamilton, 2012). However, it should be noted there were problems in this analysis resulting from a lack of viable control stimuli (e.g., neutral faces) and that much of the studies analyzed used still images as opposed to more ecologically valid moving images. In contrast, and in line with our results, recent psychophysiological findings do show an atypical response to happy faces in adolescents with autism and their siblings (Spencer et al., 2011) and individuals scoring highly on autism spectrum personality traits (Gayle et al., 2012). Specifically, Gayle and colleagues found a reduced EEG mismatch negativity response to happy but not sad images in those scoring highly on the AQ. Spencer's group found that fMRI BOLD responses to happy faces were significantly reduced compared to neutral expressions in both those with autism and their siblings but that this effect was not seen for fearful expressions; this BOLD response was observed in the fusiform face area and putative “social brain” areas, particularly the superior temporal sulcus (STS). These findings were interpreted in terms of impaired emotional reactivity in autism (Spencer et al., 2011) and argued to be consistent with diminished approach motivation and positive affect and to underlie the general negative experience of social interactions in ASD (Gayle et al., 2012). Additionally, Gayle and colleagues suggested that a reduced response to positive expression is not surprising (as it is consistent with negative social interaction), but that reduced response to negative expressions would be (as it would be consistent with positive social interaction). Our results of both decreased reactivity to happy expressions and increased reactivity to angry faces in the high AQ group fit well with this interpretation and provide even more rationale for negative social experience in ASD. The finding of increased reactivity to angry faces is also compatible with previous reports of preserved “anger superiority effect” in Asperger's syndrome (Ashwin et al., 2006).

The previous findings of decreased STS BOLD response to happy faces in ASD (Spencer et al., 2011) is interesting in relation to our present findings of decreased mu desynchronization for the high AQ group for happy faces. There is a question as to whether previous findings of decreased mu suppression to action observation in ASD reflect a problem with the core hMNS or whether it is a reflection of inefficient upstream modulation by a faulty STS (Puzzo et al., 2009). The STS can be included in descriptions of an extended hMNS (e.g., Pineda, 2008) and has been shown to be involved in several mentalizing tasks and biological motion processing (Allison et al., 2000; Spencer et al., 2011). Given that individuals with ASD show an impairment in motion perception (Dakin and Frith, 2005) and that the level of autistic traits correlates with STS structure and function (von dem Hagen et al., 2011) it is plausible to suggest that observed problems in core hMNS areas (and their associated behaviors) might stem from abnormal input from the STS (information passes from the STS to the inferior parietal lobe and then on to the inferior frontal gyrus; Pineda, 2008). This is an issue that needs to be addressed in future research.

Another issue that warrants further investigation is that of how an individual with average levels of autistic traits would react to the protocol used in this experiment. In this paper we have reported the cortical reactivity (in the form a mu ERD) of both high and low AQ scorers. We have found a strong interaction between emotional expression and AQ group, with opposite effects according to group. However, it is unknown as to whether the mu-ERD of an average AQ scorer would more resemble that of a high or low scorer or be intermediate between the two. Common sense might suggest that average scorers will be like low scorers but given that the “anger superiority effect” is also seen in typically developing individuals (e.g., Ohman et al., 2001) it is entirely plausible that the mu ERD of average scorers might resemble the pattern of results shown by high AQ scorers. In such a scenario, the findings presented here of low AQ scorers' increased mu reactivity to happy expressions and decreased reactivity (indeed ERS: event-related synchronization) to angry faces could be viewed as the more atypical reaction and might be indicative of increased empathic ability in this group. However, a recent review paper has suggested that the findings of an anger superiority effect in the general population may be an artifact of the stimuli used and that in fact, there is a tendency toward a “happiness superiority effect” (Becker et al., 2011), in which case, it is arguable that it is the low AQ group who are producing more typical responses. Clearly more work is warranted in this field, both in terms of typical and atypical development.

Another issue and possible limitation of the present study, was our use of only three emotional expressions (anger, happiness, and neutrality) with two of these (anger and happiness) being somewhat extreme. We chose not to explore other, arguably more subtle, emotions as we were primarily interested in testing the usefulness of mu-ERD in detecting individual differences in responses to emotional facial expressions. The data presented in this study goes some way to establish its value and sets the scene for further investigations into the more subtle aspects of facial processing, particularly in ASD. Other issues to be explored include, did our use of somewhat fixed facial expressions (albeit, on a moving person), influence the results. There is some evidence, for example, that individuals with ASD do better on tasks with slow dynamic facial expressions rather than static images (e.g., Gepner et al., 2001; Tardif et al., 2007). The potential for high temporal resolution in ERD/S measures puts it in a good position to answer such questions. Also, the degree to which different facial muscles are involved in different facial expressions may also have had an effect on our findings. If (as in ASD), our high AQ group was only focusing on certain parts of the faces they were presented with, then this may have had an effect on the amount of beta ERD elicited. Future work needs to investigate this possibility through the use of isolating various aspects of the expressions whilst measuring mu-suppression, preferably with the concomitant use of eye-tracking techniques.

Although not directly related to the main aims of the present study, it is also interesting to note the findings pertaining to the interaction between emotion and hemisphere in the alpha band. To recap, we found ERD to happy faces over the left hemisphere in contrast to ERS (alpha synchronization) in the right hemisphere. Additionally, we found that this ERD to happy faces in the left hemisphere was significantly different to the left hemisphere alpha activation to the angry faces (which also took the form of ERS). This suggests that hMNS activation is greater in the left hemisphere to happy faces and is intriguingly consistent with theories of hemispheric laterality in approach-avoidance actions (e.g., Maxwell and Davidson, 2007). However, at present it is unclear what alpha ERS represents in this context. It is plausible that, as in other contexts (e.g., memory and attention), alpha ERS may represent an active inhibition of cortical processing (Cooper et al., 2003; Klimesch et al., 2007) but at present this remains speculative and much more work is needed in this area to understand the possible balance between activation and inhibition in the hMNS and how this may be reflected in oscillatory activity in the mu bandwidths. What can be seen from our results as a whole, is that low beta activation may be a more sensitive index of hMNS activation than alpha. This is consistent with previous work from our lab with regard to biological motion (Puzzo et al., 2011) and extends the usefulness of this approach to the measurement of individual differences in emotional facial processing

In summary, we sought to examine the usefulness of measuring mu reactivity (changes in alpha and low beta oscillations over sensorimotor cortex) to examine individual differences in emotional facial processing. We found that those scoring highly for autistic traits had greater low beta ERD to angry than to happy faces. Those with low AQ scores exhibited the opposite pattern (greater low beta ERD to happy than angry faces) and also showed greater low beta ERD to happy faces than high scorers did. We interpret these findings in the context of the general negative experience of social interactions in ASD and propose that the measurement of mu reactivity in emotional face processing is a useful tool that facilitates the differentiation of both affective stimuli and individual differences in the level of autistic traits.

### Conflict of interest statement

The authors declare that the research was conducted in the absence of any commercial or financial relationships that could be construed as a potential conflict of interest.
